# Mining the Potential of Label-Free Biosensors for *In Vitro* Antipsychotic Drug Screening

**DOI:** 10.3390/bios8010006

**Published:** 2018-01-09

**Authors:** Tugba Kilic, Maria Soler, Nafiseh Fahimi-Kashani, Hatice Altug, Sandro Carrara

**Affiliations:** 1 Integrated Systems Laboratory (LSI), Swiss Federal Institute of Technology Lausanne, EPFL, CH-1015 Lausanne, Switzerland; tugba.kilic@epfl.ch (T.K.); sandro.carrara@epfl.ch (S.C.); 2 Bionanophotonic Systems Laboratory (BIOS), Swiss Federal Institute of Technology Lausanne, EPFL, CH-1015 Lausanne, Switzerland; maria.soleraznar@epfl.ch (M.S.); nafiseh.fahimikashani@epfl.ch (N.F.-K.); hatice.altug@epfl.ch (H.A.); 3 Department of Chemistry, Sharif University of Technology, Tehran 11155-9516, Iran

**Keywords:** label-free detection, drug screening, dopamine receptor, electrochemical biosensor, nanoplasmonic biosensor

## Abstract

The pharmaceutical industry is facing enormous challenges due to high drug attribution rates. For the past decades, novel methods have been developed for safety and efficacy testing, as well as for improving early development stages. *In vitro* screening methods for drug-receptor binding are considered to be good alternatives for decreasing costs in the identification of drug candidates. However, these methods require lengthy and troublesome labeling steps. Biosensors hold great promise due to the fact that label-free detection schemes can be designed in an easy and low-cost manner. In this paper, for the first time in the literature, we aimed to compare the potential of label-free optical and impedimetric electrochemical biosensors for the screening of antipsychotic drugs (APDs) based on their binding properties to dopamine receptors. Particularly, we have chosen a currently-used atypical antipsychotic drug (Buspirone) for investigating its dopamine D3 receptor (D3R) binding properties using an impedimetric biosensor and a nanoplasmonic biosensor. Both biosensors have been specifically functionalized and characterized for achieving a highly-sensitive and reliable analysis of drug-D3R binding. Our biosensor strategies allow for comparing different affinities against the D3R, which facilitates the identification of strong or weak dopamine antagonists via *in vitro* assays. This work demonstrates the unique potential of label-free biosensors for the implementation of cost-efficient and simpler analytical tools for the screening of antipsychotic drugs.

## 1. Introduction

Drug development is a very complex and lengthy process with an estimated 13 years from laboratory to market, including four years of preclinical research and nine years in the clinical development phase [1]. The pharmaceutical industry is facing unrivaled challenges due to high attribution rates and rising costs stemming from efficacy and safety issues [2]. Thanks to the synergy of genomics, micro-nano robotics, high-throughput screening, miniaturization, and novel analytical tools there have been significant advances in drug discovery and development [3]. Efforts have been focused on finding reliable preclinical models via the selection of appropriate animal models or 3D tissue/disease representatives with organs-on-chip platforms [4,5,6], and the identification of biomarkers and drug candidates with strong mechanisms of action via functional genomics approaches [7,8]. Of significant importance, the preclinical evaluation of drug efficacy is a very costly step and one of the most challenging in early drug development [9]. In this phase, the *in vitro* screening and analysis of drug interactions with the cellular receptors is essential not only to determine the mechanism of action, i.e., which receptor it binds, but also to quantify the affinity and obtain preliminary dose-response curves [10]. Therefore, it is necessary to establish analytical methods that enable accurate drug-receptor binding analysis, as well as the efficient screening of several drug candidates, reducing time and costs in the development process.

This demand is particularly important in the development of antipsychotic drugs. Antipsychotic drugs (APDs) are used for treatment of psychological and mental disorders, including bipolar disorder, anxiety, depression, and schizophrenia. The majority of APDs are designed in such a way that they compete with neurotransmitters, like dopamine (DA), for binding to a specific cellular receptor (i.e., G-protein coupled receptors). APDs are antagonist drugs, meaning that the binding to the receptor site impedes the interaction with the neurotransmitter and blocks the subsequent biological response [11]. However, there are at least five subtypes of dopaminergic receptors in the central nervous systems, and the signaling blockage of each type might lead to different biological responses. The first generation of antipsychotics, also known as “typical APDs”, came into being by 1950s. These APDs bind to DA type 2 (D2) receptors in mesolimbic areas and such a blockade causes some side effects, called extrapyramidal side effects (EPS) [12]. More recently, a new class of antipsychotics, “atypical APDs”, have been developed with reduced EPS profiles due to their antagonism to D3 and D4 receptors instead of D2 [13]. Therefore, careful and accurate receptor binding studies have been shown to be critical during APD development and evaluation.

The most commonly used *in vitro* receptor-based APD screening techniques are DA receptor autoradiography [14], radio-ligand binding assays with some commercialized kits such as SPA (GE Healthcare, Little Chalfont, UK, or PerkinElmer, Hong Kong, China), LanthaScreen™ system (Invitrogen), or fluorescence polarization-based cAMP kits (PerkinElmer, Molecular Devices, Sunnyvale, CA, USA, and GE Healthcare), and immunoblotting for determination of Fos protein expression [15]. These techniques make use of radioactive or fluorescent labels to monitor the response after target-receptor binding [10,16]. However, labeling is not only a tedious and costly procedure, but it also generally reveals several problems due to false positives because of background binding of hydrophobic fluorescent labels, and false negatives because of binding site occlusion. Therefore, label-free, sensitive, and preferably high-throughput screening methods are required. On this behalf, biosensors provide unique solutions as they are able to give information about drug candidate-receptor interaction in a fast and user-friendly manner. Among others, especially optical biosensors and electrochemical biosensors have shown great promise for label-free screening of neurotransmitters and drug candidates [17,18]. Until now, these biosensors have been employed only for detection and quantification of either antipsychotic drugs [19,20,21] or dopamine [22,23,24,25,26] as isolated assays, so the design of functional drug-receptor *in vitro* binding assays providing competitive inhibition information has not been fully accomplished yet with nanoplasmonic biosensors and impedance-based biosensors. 

In this paper, for the first time, we have investigated the potential of two label-free platforms for receptor-based APD screening: an electrochemical biosensor based on electrochemical impedance spectroscopy (EIS), and a nanoplasmonic sensor based on spectroscopic imaging. The working principle of these biosensors basically consists in the detection of molecular interactions happening on the transducer surfaces (i.e., carbon screen-printed electrodes, CSPE, and gold nanohole arrays, AuNHA, respectively) directly by monitoring the changes induced in the electrical signal within a circuit or the transmitted light spectra, respectively. In a previous work, Kilic et al., have studied the interaction of APDs with dopamine D3 receptor peptide (D3R) with an electrochemical biosensor based on voltammetry. They demonstrated that the binding of APDs to the D3R could be detected through changes in the oxidation signal [27]. Here, we have designed a competitive dose-response inhibition assay for the evaluation of APD affinity for the dopamine D3 receptor using both label-free biosensors. A currently used atypical APD (i.e., Buspirone) has been chosen as a model, and the competitive antagonist effect is evaluated versus dopamine and negative control drugs. Despite the different sensing transduction mechanism and materials, we have applied similar surface functionalization and assay schemes, which allow for comparing and evaluating the capabilities and analytical features of both biosensors. Efforts have been directed to achieve not only highly sensitive detection of the drug and dopamine, but also to ensure the selectivity and provide reliable dose-response inhibition curves for the evaluation of receptor binding affinities. With this work, we demonstrate the possibility of implementing label-free biosensor-based APD screening assays and open up new means to improve and simplify the process of drug discovery and development.

## 2. Materials and Methods

### 2.1. Reagents and Apparatus

Carbon screen-printed electrodes (CSPE), DRP-110, used for EC sensing purposes, were bought from DropSens (Llanera, Asturias, Spain). EC measurements were conducted by an AUTOLAB PGSTAT204 (Metrohm AG, Herisau, Switzerland) compact and modular potentiostat/galvanostat. Gold nanohole arrays (AuNHA) were fabricated in-house as described elsewhere [28]. Optical measurements were performed with an inverted microscope (Nikon Eclipse-Ti, Tokio, Japan) and a CCD spectrometer (Andor Shamrock 303i, Andor Technology Ldt., Belfast, UK).

All chemicals used throughout the study; phosphate-buffered saline (PBS, 1×), K_4_Fe(CN)_6_, K_3_Fe(CN)_6_, KCl, dopamine hydrochloride, buspirone hydrochloride, etoposide, and acetaminophen (APAP), carboxylic acid-functionalized 50 nm gold nanoparticles coated with polyethylene glycol (PEG 3000), crosslinking reagents EDC, sulfo-NHS, bis(sulfosuccinimidyl)suberate (BS3), ethanolamine, bovine serum albumin (BSA), and Tween 20 were purchased from Sigma-Aldrich (Buchs, Switzerland). PEGylated alkanethiol compounds HS-C_6_EG_4_OH and HS-C_11_EG_4_OCH_2_COOH were acquired from Prochimia Surfaces (Sopot, Poland). Dopamine (D3) receptor synthetic peptide (ab128688) was purchased from Abcam (Cambridge, UK).

### 2.2. Electrochemical Impedance Spectroscopy (EIS) Measurements

Disposable carbon screen-printed electrodes (CSPE) were used for EIS measurements. CSPE 3.4 × 1.0 × 0.05 cm consist of a carbon working electrode (4 mm diameter), a silver reference electrode, and a carbon counter electrode. EIS measurements were performed with an Autolab potentiostat at the following instrumental settings: set potential (V) = 0.101, frequency range (Hz) = 10^1^–10^5^, and AC amplitude (V) = 0.005. For the analysis, the working electrode is functionalized and incubated with the analyte sample, and electrochemical impedance is measured. Nyquist plots were drawn based on the Randles model, which is based on solution resistance (R_s_), charge transfer resistance (R_ct_), Warburg impedance (W), and double-layer capacitance (C_dl_). Nova 1.11 software was used for simulation of the data and the electrochemical circle fit option was chosen to simulate the experimental results according to the Randles model. Charge transfer resistance (R_ct_) was analyzed after each assay step. All calibration curves were drawn based on normalized R_ct_ values, which is defined as NR_ct_ = (R_ct_)_D3R + ligand_/(R_ct_)_BSA-DA conjugate_. Ligand, in this definition, it is referred to either DA or the drug based on the measurement.

### 2.3. Nanoplasmonic Spectroscopic Imaging Measurements

Plasmonic nanohole arrays (NHA) were fabricated via a lift-off-free wafer-scale fabrication scheme based on deep-UV lithography [28]. Each plasmonic chip (1 cm × 1 cm) is composed of eight independent nanohole arrays (3 × 3 matrix). Each NHA has an area of 100 × 100 μm (200 nm hole diameter, 600 nm array periodicity). For spectroscopic measurements, the chip was integrated into a multichannel microfluidic system, including three sensor arrays per channel. The chip is illuminated with normal broadband light and the transmitted optical response is acquired from the three sensor arrays simultaneously. The transmitted light is coupled to a CCD spectrometer where each NHA is spread as a single spectrum (wavelength 850 nm, 0.5 s exposure time). The different spectra are independently extracted and analyzed with a homemade MATLAB script to provide monitoring of the EOT peak shifting. The peak position is determined by calculating the centroid of the peak within a fixed wavelength window (40% maximum intensity) and plotted in real-time (i.e., sensorgrams). Calibration curves were obtained by plotting the normalized EOT peak shift values (i.e., resonance shift) for each analyte concentration. Curves were fitted to a dose-response inhibition equation for extracting the LOD (90%) and IC50 values.

### 2.4. Synthesis of DA-BSA Conjugate and Surface Functionalization

BSA was used as carrier protein for DA-BSA conjugate. Synthesis was performed according to well-established conjugation protocols [29]. Briefly, BSA was first incubated with 10× molar excess of crosslinker molecule bis(sulfosuccinimidyl)suberate (BS3) in PBS 1× for 1 h at room temperature (RT) and gentle agitation. After, BS3-modified BSA was purified by filter centrifugation (Amicon Ultra, 30 kDa, Sigma-Aldrich) and rapidly mixed with 10x molar excess of dopamine hydrochloride (DA). The mixture was incubated for 2 h in the dark at room temperature and gentle agitation. Finally, the DA-BSA conjugate was again purified by filter centrifugation and reconstituted to 1 mg/mL solution in PBS. The conjugate was aliquoted and stored at −20 °C.

Both sensor surfaces were functionalized with DA-BSA conjugate for capturing of D3R, but different functionalization protocols were applied according to the surface material. For the EC biosensor, 5 μg/mL DA-BSA was drop casted on the CSPE for 2 h at 4 °C in the dark for physical adsorption. After, the sensor surface was rinsed with PBS. For optical measurements, the gold surface was chemically modified to achieve covalent attachment of DA-BSA. Briefly, a mixed PEGylated self-assembled monolayer (COOH:OH 1:5, 1 mM) was created by incubation overnight in ethanol at RT. After rinsing, COOH groups were activated via carbodiimide chemistry (EDC 400 mM, s-NHS 100 mM) in MES buffer for 20 min, and readily incubated with 50 μg/mL DA-BSA in PBS overnight at 4 °C.

### 2.5. D3 Receptor Binding Assay Procedure

The D3R used in this study has the peptide sequence of CQACHVSPELYRATTWGY. This sequence corresponds to the amino acids 402–419 in mouse and rat and amino acids 356–373 in human. It is related to the last extracellular loop of the dopamine 3 receptor. As it is done in similar studies [30,31], we also used the synthetic peptide instead of the whole receptor protein because of the higher stability of the peptides.

For EC measurements, D3R was preincubated with DA and buspirone samples at different concentrations. Incubation was carried out at 4 °C in the dark for 1 h and then the sample was drop casted on the functionalized CSPE for 1 h. After, the surface was washed with PBS and measurements were taken in redox solution (pH: 8.0) prepared by 2 mM K_4_Fe(CN)_6_, 2 mM K_3_Fe(CN)_6_, and 46 mM KCl in PBS. 

For optical measurements, D3R was previously attached to gold nanoparticles in order to increase the relative refractive index change and improve the detection efficiency. For that, we employed 50 nm COOH-functionalized gold nanoparticles (AuNPs) and D3R coupling was achieved via EDC/s-NHS chemistry. Briefly, AuNPs were incubated with 150 mM EDC and 50 mM s-NHS solution in MEST buffer (10 mM, pH 5.4, 0.05% Tween 20) for 30 min. Then, activated AuNPs were vortexed thoroughly with PBST 0.1× (0.05% Tween 20) and separated by centrifugation. After discarding the supernatant, NHS-terminated AuNPs were incubated with 100 µg/mL D3R in PBS 0.1× at 4 °C for 2 h. The resultant mixture was vortexed thoroughly with PBST 0.1× and centrifuged to discard free D3R. Finally, D3R functionalized AuNPs were blocked with BSA 0.5% solution for 30 min at 4 °C, purified, and dispersed in PBS 0.1×. Each AuNP was calculated to contain a maximum of 11 D3R molecules. Similar to EC measurements, D3R-AuNPs were incubated at a fixed concentration with DA and buspirone at 4 °C in the dark for 30 min, and then introduced to the microfluidics-integrated functionalized nanoplasmonic chip. Capture of non-reacted D3R-AuNPs was monitored for 30 min (flow rate 30 μL/min). After each sample measurement, the functionalized surface was regenerated with NaOH 20 mM solution (flow rate 50 μL/min) and it can be reused for at least 10 measurements.

In both cases, data were analyzed using GraphPad Prism 7.03 software. Statistical analysis was performed as one-way analysis of variance (ANOVA) with the statistical significance threshold set at 0.05.

## 3. Results

### 3.1. Design of Biosensor Strategies for D3R Competitive Inhibition Assay for APD Screening

To evaluate the potential of biosensors for *in vitro* receptor-based APD screening, we selected two well-known sensing platforms with demonstrated superior capabilities for performing label-free biochemical analysis. In particular, we employed an electrochemical sensor based on impedance spectroscopy and a nanoplasmonic sensor based on spectroscopic imaging.

**Figure 1:**
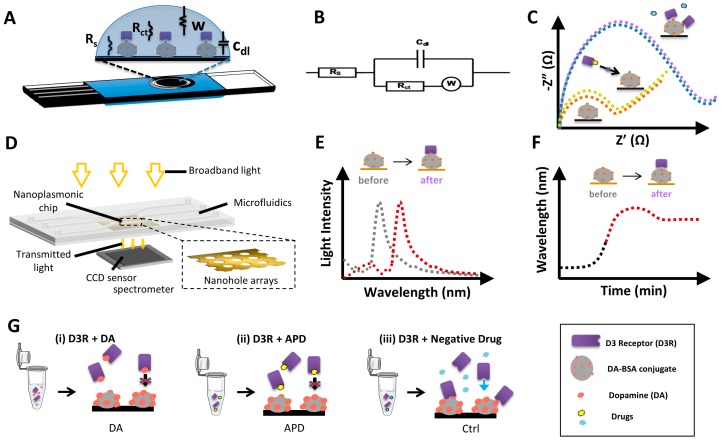
Conceptual illustration of the label-free biosensors working principle and drug screening assay design. (**A**) Schematics of the electrochemical sensor based on carbon screen-printed electrodes (EPS). (**B**) Randles equivalent circuit for an electrode in electrolyte contact. (**C**) Illustrative representation of electrochemical impedance spectroscopy (EIS) signals, before and after interaction of the DA-BSA conjugate with D3R for the case of APD or control drug interactions. (**D**) Schematics of the nanoplasmonic sensor based on nanohole arrays. (**E**) Illustrative representation of extraordinary optical transmission (EOT) spectroscopy signal, before and after analyte capture. (**F**) Illustrative representation of the real-time sensorgram extracted from spectroscopic displacements during the analyte capture. (**G**) Schematics of the APD screening assay based on indirect competitive detection: (i) Standard: dopamine binds to D3R in solution and impedes its binding to the DA-BSA conjugate; (ii) A good antipsychotic drug (APD) will also bind to D3R and hamper its binding to the DA-BSA conjugate; and (iii) a non-specific drug will not bind to D3R and it will bind to the DA-BSA conjugate.

Electrochemical impedance spectroscopy (EIS) is a technique used to measure electrical changes on the electrode surface due to receptor-ligand binding and often used as a label-free detection strategy for immunosensors [30,32]. The mechanism of detection is based on the change of interfacial electron-transfer kinetics of the redox probe [Fe(CN)_6_]^4−/3−^ upon changes on electrode surface due to receptor binding and receptor-ligand interaction. In the current study, we use D3R as the receptor and either DA or buspirone as ligands to be able to detect the changes on electrode surface via Nyquist plots represented by Figure 1C. The charge transfer resistance R_ct_ of the redox probe was determined via simulation of the raw data by Nova software based on the equivalent circuit as represented by Figure 1B, composed of the circuit elements; R_ct_ ; solution resistance, R_s_; double-layer capacitance, Cdl; and Warburg impedance, W. Carbon screen-printed electrodes (CSPE) composed of three electrodes: the working electrode, where the biorecognition reaction occurs, a reference electrode, and the counter electrode that provides the voltage (Figure 1A).

In label-free nanoplasmonic sensing, we measure changes in the optical properties of the light upon interaction with a nanostructured metallic surface (e.g., gold). The light coupling to the plasmonic structures generates the so-called surface plasmon resonances, which are characterized by a series of peaks and dips in the light spectrum that are highly sensitive to refractive index changes. Among the different plasmonic sensing schemes, we particularly employed gold nanohole array chips that exhibit the extraordinary optical transmission (EOT) resonance. EOT occurs when a highly ordered array of plasmonic nanoholes is illuminated at normal incidence, originating a resonance peak in the transmitted spectra (Figure 1D). When a biomolecular interaction takes place on the nanohole array, the increase in mass produces a change of the refractive index of the medium, which, in turn, induces a shift of the EOT resonance peak (Figure 1E). Continuous interrogation of the EOT peak position allows the real-time acquisition of sensorgrams that monitor the molecular binding in a label-free manner (Figure 1F) [28,33]. Furthermore, in order to perform real-time analysis, our plasmonic chip was integrated into a microfluidic system that facilitates sample delivery to the sensor arrays in a simple and user-friendly manner. We employed a three-channel microfluidic system, with each channel covering three independent sensor arrays, so we were able to obtain simultaneous replicates, as well as include reference control sensors if needed.

An *in vitro* assay for APDs-receptor binding analysis generally consists of measuring the competitive efficacy of the drug for binding to the receptor, compared to the natural neurotransmitter dopamine (DA). In this regard, we have designed a standard competitive inhibition assay in which a DA-BSA conjugate is immobilized on the sensor surface as the capture agent, and the D3R is used as a biorecognition element in solution. Samples containing the target analyte (i.e., DA or APD) are incubated with the D3R and, thereafter, exposed to the functionalized sensor surface. A priori, both DA and APD would interact with the receptor, occupying the binding sites, so that the D3R will not attach to the functionalized sensor surface (Figure 1G). However, if the receptor is incubated with a nonspecific control drug, the D3R binding sites are free to attach to the DA molecules functionalized on the sensor surface. Finally, by comparing the signals obtained with the drug to the natural neurotransmitter DA we could predict whether the APD will interact with the cellular receptor and estimate, qualitatively, the relative affinity.

### 3.2. Electrochemical Biosensor for APD Drug Screening

#### 3.2.1. Characterization of the Electrochemical Biosensor

**Figure 2:**
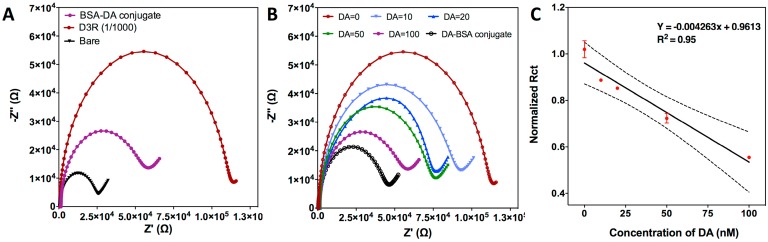
Characterization of EC biosensor for indirect DA detection. (**A**) Nyquist plots drawn for different stages of functionalization of CSPE; bare, BSA-DA conjugate-immobilized and D3R (dilution: 1/1000). (**B**) Indirect detection of DA at various concentration; 0, 10, 20, 50, 100 nM. Color code: D3R: bordeaux; bare: black; BSA-DA conjugate: purple. (**C**) Calibration curve of EC biosensor for indirect DA detection based on normalized R_ct_ values. Each value represents the mean and SD of five measurements.

As a first step, characterization of the electrochemical sensor was done by looking at CSPE surface functionalization efficiency and D3R detection. The carbon surface was modified by physical adsorption of DA-BSA conjugates. This functionalization strategy is based on drop-casting of the sample on the working electrode surface, incubating for 2 h at 4 °C and washing the unbound molecules. EIS measurements were performed for bare CPSE and after DA-BSA conjugate immobilization, which produced an increase of the R_ct_ value from 22.86 kΩ to 65.95 kΩ (Figure 2A). The measurements were repeated with five electrodes at the same conditions to confirm the reproducibility of the procedure (data not shown). Then, the functionalized chips were incubated with D3R (1/1000 dilution factor) to evaluate the capture efficiency of the DA-BSA conjugate. D3R attachment caused a significant increase in R_ct_ value up to 113.00 kΩ due to the insulating effect of the D3R peptide on the CSPE surface (Figure 2A). The signal obtained for pure D3R was high enough to be our blank signal and analyze the effect of the dopamine on it.

After showing that CSPE is properly functionalized and D3R can be detected due to its interaction with the BSA-DA conjugate, indirect DA detection studies were performed. Various DA concentrations ranging from 0 to 100 nM were mixed with D3R and incubated for 1 h. A mixture of DA-D3R was then drop-casted on BSA-DA-conjugated CSPE for another incubation of 1 h. The change in R_ct_ after such interaction has shown that increasing the amount of DA in the mixture decreased the free D3R levels and, therefore, resulted in less DA-BSA interaction with D3R. This can be seen in Figure 2B where Nyquist plots at higher DA concentration show lower R_ct_ values due to reduced free D3R amount. With these values, we could obtain a dose-response curve for indirect DA detection (Figure 2D). When the DA concentration reached 100 nM, the R_ct_ reached almost to the R_ct_ of BSA-DA conjugate. A further increase of DA concentration resulted in non-specific binding/adsorption of unoccupied DA to the electrode surface and, hence, resulted in impedance changes as reported in other studies [34]. Therefore, with the given D3R concentration, EIS was able to indirectly detect DA up to 100 nM. The calibration curve drawn for indirect DA detection showed a linear tendency. The limit of detection (LOD) of the sensor LOD = 3.3 σ/S (where σ is the standard deviation of the DA-BSA conjugate and S is the slope of the calibration curve of Dopamine) [32] as 2.87 ± 0.25 nM. 

#### 3.2.2. Competitive Inhibition Assay for D3R-Antagonist Antipsychotic Drug: Detection of Buspirone and Specificity Controls

**Figure 3:**
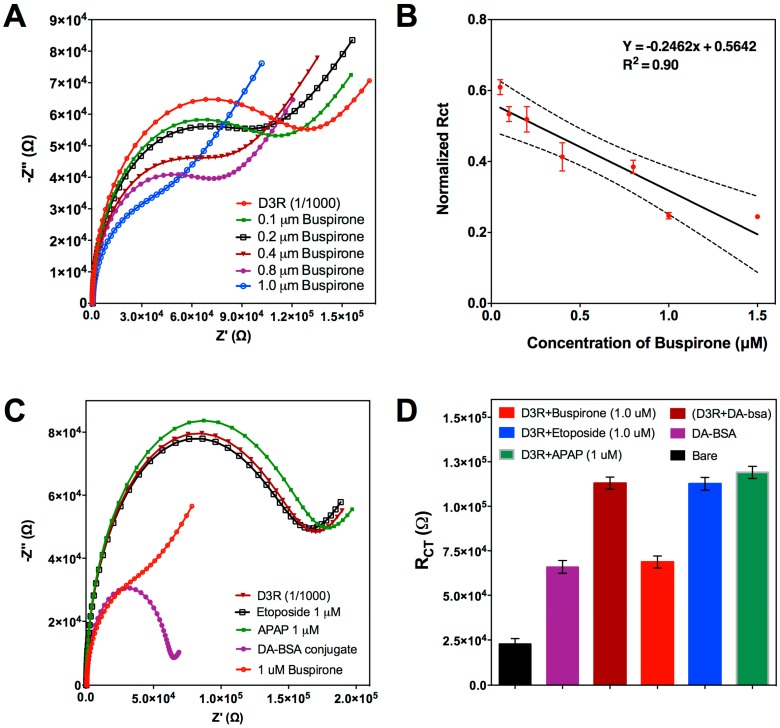
Electrochemical inhibition assay for buspirone. (**A**) Nyquist plots drawn for various buspirone concentrations (0.1, 0.2, 0.4, 0.8, 1.0 μM). (**B**) Calibration curve of EC biosensor for indirect buspirone detection based on normalized R_ct_ values. Each value represents mean and standard deviation of five measurements. (**C**) Nyquist plots drawn for control studies performed with Etoposide and APAP at the same concentration of buspirone (1.0 μM). (**D**) Bar graphs with mean and SD (*n* = 5) for signal comparison between control drugs and buspirone at the same concentration (1.0 μM).

After indirect detection of DA, we investigated the potential of the designed biosensor for indirect buspirone detection. The assay procedure was performed as explained in Section 3.2.1., but instead of DA, the D3R was mixed with different concentrations of buspirone (0.1–1 μM). Similar to the previous findings, higher buspirone concentration was shown to be decreasing R_ct_ signals after interaction with the BSA-DA conjugate on the CSPE surface (Figure 3A). Compared to Figure 2B, Figure 3A has different Nyquist plots with a more pronounced Warburg impedance. This change could be attributed to the fact that buspirone HCl has a larger molecular weight (385.5 g/mol) then DA (153.18 g/mol), therefore, the complex they have with D3R have different structures; assumedly buspirone-D3R would occupy more space inside the solution and, hence, it would be more difficult for free D3R to diffuse to the sensor surface and increase the Warburg impedance. At the 1 μM buspirone concentration, the R_ct_ signal reached the R_ct_ of BSA-DA. Therefore, 1 μM buspirone is thought to be enough for D3R saturation. That result also showed that, like DA, buspirone also has a D3R binding property. The calibration curve drawn for different Buspirone concentration versus normalized R_ct_ (Figure 3B) showed a linear tendency again as expected. The LOD was calculated for buspirone as 0.12 μM.

The analytical selectivity and reproducibility were also investigated in order to assure the accuracy and reliability of the assay. Negative control studies were performed by using etoposide (cancer treatment) and acetaminophen (APAP, common pain treatment), thus, they should not bind to D3R. Two samples containing 1 μM of each drug were incubated with D3R peptide following the same conditions established for the APD and DA, and the mixture was then measured with the DA-BSA-functionalized CSPE. As can be seen in Figure 3C, neither of the drugs reacted with the D3 receptor and the R_ct_ signals obtained are equal to the response of pure D3R. This experiment confirms that D3R only binds to either specific APDs or the neurotransmitter DA, so the signal response can be exclusively attributed to a specific interaction with target analytes. To demonstrate the reproducibility of the biosensing assay, the whole experiments were repeated with five CSPEs, obtaining a coefficient of variation (CV = standard deviation/mean) of 3.1–5.8%, which is below the maximum value permitted for analytical techniques (i.e., 15%) [35]. It is worth mentioning that the R^2^ values both for DA and buspirone detection is lower than 0.99. We could attribute this to the fact that EIS is very sensitive to the changes on the electrode surface, and free DA and buspirone could adsorb to the electrode surface and change the impedance values.

### 3.3. Nanoplasmonic Biosensor for APD Drug Screening

#### 3.3.1. Characterization of the Nanoplasmonic Biosensor

**Figure 4:**
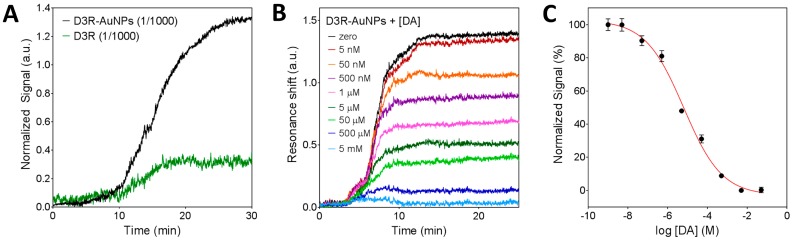
Characterization of the nanoplasmonic biosensor for indirect DA detection. (**A**) Sensorgrams obtained for the detection of pure D3R and gold nanoparticles conjugated D3R (D3R-AuNPs) on the DA-BSA functionalized surface. (**B**) Sensorgrams obtained for indirect detection of dopamine at different concentrations (5 nM–5 mM). (**C**) Standard dose-response inhibition curve for indirect detection of DA. Plots represent mean and standard deviation of each DA concentration (1 nM–5 mM) measured in triplicate.

Unlike impedance measurements that directly depend on the isolating response of molecules on the electrode, the nanoplasmonic signal is directly related to the change of mass on the chip and a subsequent change of refractive index. In this regard, the D3R peptide is a rather small molecule, so we would need high concentrations to achieve significant sensor signals. In order to maximize the biosensor efficiency, as a previous step, the D3R peptides were conjugated to gold nanoparticles (D3R-AuNPs). The use of metallic nanoparticles helps to induce a larger effective change of the refractive index due to the different material. For the conjugation, we employed COOH-modified 50 nm AuNPs and covalently attached the D3R peptide through the carbodiimide chemistry (EDC/NHS). This strategy has been shown to greatly enhance the sensor signal without affecting the biomolecule activity and target recognition [36]. On the other hand, for the plasmonic measurements, the chip is assembled in a microfluidic system for the in-flow delivery of the sample on the functionalized surface. Therefore, simple physical adsorption of the DA-BSA conjugate on the surface would not be stable enough and thus compromising the analysis reproducibility. Instead, we took advantage of the well-known gold surface chemistry and applied a functionalization protocol for the covalent attachment of the DA-BSA conjugate to the sensor. Briefly, we created a PEGylated self-assembled monolayer (SAM) with functional COOH-ending groups. By means of the carbodiimide activation chemistry (EDC/NHS), we immobilized the DA-BSA conjugate through a highly stable amide bond and, finally, deactivate the surface with ethanolamine. In order to test the functionalization efficiency, we introduced a D3R-AuNPs sample at 1/1000 dilution ratio. As can be seen in Figure 4A, D3R-AuNPs attaching to the DA-BSA conjugate induced a significant resonance shift. By comparing the signal obtained with the nanoparticles to the signal induced by non-conjugated D3R (1/1000 dilution ratio), we clearly demonstrate the significant enhancement achieved with the AuNP conjugation. The signal obtained for the capture of D3R-AuNPs was high enough to be established as a blank signal for the competitive inhibition assay.

Once the biosensor functionalization was characterized, we evaluated the sensitivity for indirect DA detection. The D3R-AuNPs were incubated with different concentrations of DA (1 nM to 5 mM) for 30 min and thereafter introduced in the biosensor. Figure 4B shows the obtained sensorgrams corresponding to free D3R binding to the DA-BSA surface. As it can be seen, increasing the concentration of DA resulted in lower D3R-AuNPs signal until all D3R binding sites were occupied (5 mM) and no signal was obtained. By plotting the normalized sensor signals as a function of DA concentration (in logarithmic scale), we obtained a typical dose-response inhibition curve (Figure 4C). The limit of detection was determined as 5 nM and the IC50 value was 6.4 ± 0.7 μM. It is worth mentioning that the IC50 value determined in our assay refers to the concentration corresponding to 50% of the signal and due to inherent and crucial differences with an actual biological system; the value cannot be taken as the pharmaceutical IC50, but only a relative analytical parameter to compare different analytes within the same biosensing assay conditions.

#### 3.3.2. Inhibition Assay for D3R-Antagonist Antipsychotic Drug: Detection of Buspirone and Specificity Controls

**Figure 5:**
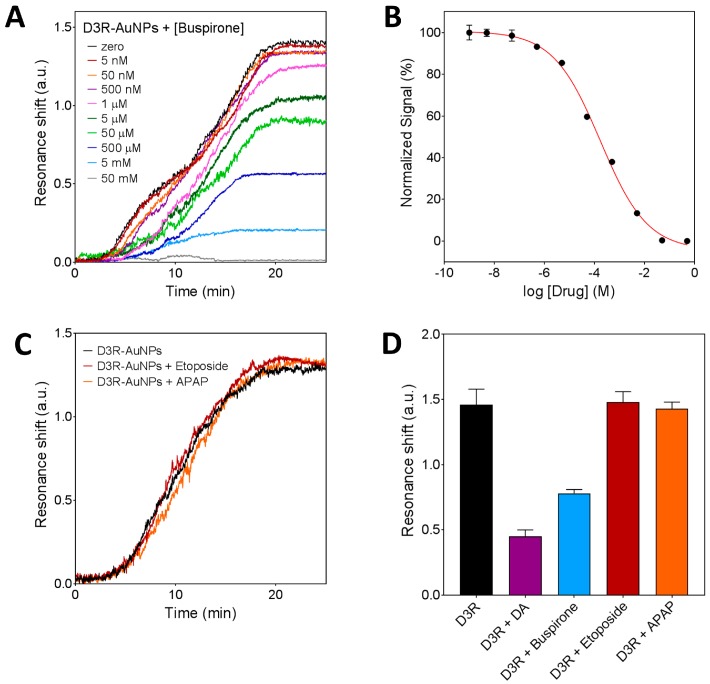
Nanoplasmonic inhibition assay for D3R-antagonist antipsychotic drug. (**A**) Sensorgrams obtained for indirect detection of buspirone at different concentrations (5 nM–5 mM). (**B**) Standard dose-response inhibition curve for indirect detection of Buspirone. Plots represent mean and standard deviation of each buspirone concentration (1 nM–5 mM) measured in triplicate. (**C**) Sensorgrams obtained for negative control drugs (i.e., etoposide and APAP), compared to pure D3R-AuNPs detection. (**D**) Signal comparison between dopamine, antipsychotic drugs, and control drugs at the same concentration (6.5 μM). Statistical analysis (*n* = 5) confirmed that differences between control drugs signals and free D3R are not significant (*p* < 0.05).

In order to analyze the antagonist effect of buspirone as APD for D3 receptor, we carried out the inhibition competitive assay using samples with different drug concentration (5 nM–5 mM) and following the same conditions as before. Figure 5A shows the real-time sensorgrams obtained for free D3R binding after drug incubation. It can be seen that increasing concentrations of buspirone caused a decrease of the sensor signal, confirming that buspirone also interacts with the D3R, similar to DA. It is worth mentioning that the sensorgram traces for the buspirone assay appear to be slightly different to the ones obtained for DA (Figure 4B). For the DA case, the binding reaches equilibrium faster than in the buspirone assay. Although we cannot confirm, a hypothetical explanation would be that D3R-AuNPs approach the surface with a majority of the binding sites occupied by buspirone. However, when contacting the DA molecules present on the sensor surface, buspirone is displaced due to a higher affinity of the D3R for the natural neurotransmitter. We also obtained the corresponding inhibition dose-response curve and we determined the limit of detection at 500 nM concentration. The IC50, in this case, was found at 430 ± 14 μM. The buspirone IC50 value, being higher than the DA IC50 (6.4 μM), confirms that, according to the literature [37], buspirone is an antagonist for D3R, but presents lower affinity than the natural neurotransmitter.

Finally, to prove the reliability of the assay we evaluated the selectivity and reproducibility of the biosensor strategy. As we did for the EC measurements, two samples of negative control drugs (etoposide and APAP) were incubated with the D3R-AuNPs and the mixture was then delivered onto the DA-BSA functionalized chip (Figure 5C). Signals obtained for both drugs at 5 mM concentration were equal to the signal obtained when using free D3R-AuNPs, which indicates that the peptide only reacts with either DA or buspirone, and the biosensor response is not influenced by the presence of other drugs or molecules in the sample. We performed a statistical analysis to compare the signals obtained by the different analytes at the same concentration (6.5 μM, the DA IC50 value) among them and to the free D3R. As can be seen in Figure 5D, differences between free D3R and control drugs were not significant (*p* < 0.05), while a clear difference was observed when incubating with DA and buspirone. This result also suggests that a one-step assay (employing a concentration close to the IC50 obtained for the DA) could be implemented for fast drug screening. The reliability and reproducibility of the nanoplasmonic sensing assay were also confirmed by repeating the experiments with five different chips obtaining a coefficient of variation of 8%.

## 4. Discussion and Conclusions

The implementation of novel analytical techniques for *in vitro* receptor binding assays is in urgent demand for improving the drug discovery and development process. Current techniques, mostly based on labeling methods, are time-consuming, expensive, and they usually lead to inaccurate results (REFs). Particularly for antipsychotic drug (APD) discovery, the implementation of new receptor-ligand assay techniques that enable a more efficient screening and evaluation of drug candidates is in urgent demand. In this regard, label-free biosensors might offer an interesting alternative as simple, integrable, and fast analytical platforms. 

Here, we have demonstrated the unique potential offered by label-free biosensors as integrable and efficient tools for antipsychotic drug screening and analysis. We have focused on two well-known label-free biosensing principles: electrochemical impedance spectroscopy and nanoplasmonic spectroscopic imaging. These biosensors have been applied to APD screening and evaluation by analyzing the competitive antagonist effect in the D3 dopaminergic receptor. We propose a novel analysis strategy based on standard competitive inhibition assays, where a DA-BSA conjugate is immobilized on the sensor surface, and D3R is used as a specific biorecognition element in solution for the indirect detection of the APD. By analyzing the dose-response curve in comparison to dopamine binding, we can evaluate the affinity and predict the antagonist effect of the drug for the cellular receptor. Recent works in the field have proven the highly-sensitive detection of either the drug or the neurotransmitter (i.e., dopamine, DA) employing label-free biosensors. For example, Feng et al., introduced a three-dimensional graphene biosensor for the detection of dopamine reaching a limit of detection of 1 nM [38]. Ben-Yoav et al., have shown the in situ detection of an antipsychotic drug (i.e., clozapine) in serum with the required effective range, proposing the biosensor for the point-of-care therapy monitoring [19]. On the other hand, plasmonic sensors, such as the well-known surface plasmon resonance (SPR) systems, have been applied for direct dopamine detection, achieving sensitivities in the pM range [30]. However, these methodologies did not analyze the intrinsic competition between the drug candidate and the neurotransmitter for the cellular receptor occupancy, which is crucial information in the drug discovery process. Furthermore, our biosensors offer exceptional capabilities in terms of integration and multiplexing that are crucial for their implementation as a routine analysis technique in pharmaceutical laboratories.

For impedimetric sensing, we employed the widely used commercial CSPE that has shown excellent reliability and reproducibility. The label-free EC assay is extremely sensitive, easy to perform, and does not require any sophisticated surface chemistry procedures, which inherently implies minimum costs and a relatively simple implementation. For the nanoplasmonic sensor, we selected plasmonic nanohole arrays, which can be produced at a large scale using conventional and cost-effective nanofabrication techniques. Unlike the traditional SPR biosensor that requires complex prism-coupling schemes, our nanoplasmonic biosensor works with normal-incident broadband light, therefore enabling the integration in customized microfluidics lab-on-a-chip devices that can be used in common inverted microscopes [33]. Furthermore, the multiplexing capabilities of nanohole arrays could be exploited for the parallel analysis of different drugs, dramatically reducing the analysis time and cost [28].

In terms of sensitivity, we reached limits of detection at the nM level, which enables performing the APD screening at concentrations similar to the physiological levels in the brain. Regarding the linear range, we observed that D3R saturation occurs at lower concentrations in the case of EC sensors (0.1–1 μM) while, for the nanoplasmonic sensor, the detection range extends to mM concentrations (5 nM–5 mM). We could attribute this to a larger amount of either D3R molecules in the solution or DA-BSA binding sites on the nanoplasmonic biosensor. It is worth mentioning though that due to differences in the sensing principle, the EC biosensor was more sensitive for pure D3R peptide capture, while the nanoplasmonic biosensor required a previous conjugation to gold nanoparticles to be able to use relatively low concentrations of D3R and reduce costs. In terms of selectivity, our biosensors demonstrated a high specificity for detecting DA or APDs, with no interference from other drugs. This is especially important since we employ label-free affinity-based biosensors and the nonspecific binding of molecules to the sensor surface could lead to false positive signals. However, the design of an indirect detection assay, the use of a highly-specific receptor biomolecule, and a correct surface functionalization ensured the selective detection of target analytes. Finally, we also tested the analytical reproducibility of the biosensors obtaining, in both cases, variation coefficients well below the maximum established for clinical analytical techniques (15%).

In this paper, we have demonstrated the unique potential of label-free biosensors for a simple, reliable and rapid screening and analysis of antipsychotic drugs. This work opens new avenues for the investigation and implementation of innovative analytical methods in the pharmaceutical industry that reduce costs and improve drug discovery.
